# “The Power of Ethical Leadership”: The Influence of Corporate Social Responsibility on Creativity, the Mediating Function of Psychological Safety, and the Moderating Role of Ethical Leadership

**DOI:** 10.3390/ijerph18062968

**Published:** 2021-03-14

**Authors:** Byung-Jik Kim, Min-Jik Kim, Tae-Hyun Kim

**Affiliations:** 1 College of Business Administration, University of Ulsan, Ulsan 44610, Korea; kimbj82@business.kaist.edu; 2 School of Industrial Management, Korea University of Technology and Education, 1600, Chungjeol-ro, Chungcheongnam-do 31253, Korea; mkim@koreatech.ac.kr; 3 College of Business, Korea Advanced Institute of Science and Technology, Seoul 02455, Korea

**Keywords:** corporate social responsibility (CSR), employee creativity, employee psychological safety, ethical leadership, moderated mediation model

## Abstract

A body of existing literature delves into how corporate social responsibility (CSR) affects employees’ cognition, emotion, and behavior within an organization. These previous studies, however, pay relatively little attention to the influence of CSR on levels of creativity in employees. Considering that creativity is closely related to innovative capability, which is critical for a firm to survive, the relationship between CSR and employees’ creativity and its elaborate underlying processes need further investigation. Based on a group creativity model, we argue that CSR may increase levels of creativity in employees through mediation of enhanced levels of psychological safety in employees. In addition, existing works on CSR have relatively underexplored the contextual role of leadership in translating CSR practices into employees’ attitudes, perceptions, and behaviors. Using three-wave time-lagged survey data from 311 employees in South Korea, we found that CSR enhances employees’ creativity via mediation of psychological safety. Additionally, ethical leadership positively moderates the relationship between CSR and psychological safety. Our findings suggest that psychological safety in employees functions as an important underlying mechanism to describe the CSR–employee creativity link. Furthermore, this paper emphasizes the importance of the moderating role of ethical leadership in the process of CSR activities.

## 1. Introduction

Corporate social responsibility (CSR) refers to the efforts of firms to enhance the welfare of various stakeholders such as shareholders, employees, customers, local communities, and the environment in operating a business [1,2,3,4,5]. Many previous studies have found that CSR functions as a strategic resource to facilitate competitive advantages in companies [6,7,8]. For example, CSR increases a variety of dimensions of organizational effectiveness, including customer evaluations of the firm and its products, corporate reputation, employees’ positive reactions, and financial performance [4,6,8,9,10,11].

Despite much evidence showing the association between CSR and a variety of organizational outcomes, research gaps remain that need to be adequately addressed. First, previous works have paid little attention to the association between CSR and organizational outcomes at the micro-level, including employees’ perceptions, attitudes, and behaviors in an organization. Extant studies have mainly examined the impact of CSR on macro-level outcomes such as financial performances, product quality, and corporate reputation by focusing on external stakeholders including shareholders, customers, and local communities [12,13,14,15]. Considering that employees are important internal stakeholders who plan, initiate, and implement CSR practices in the first place [13,14,15], investigation of the reactions of employees toward CSR activities—that is, the micro-foundation of CSR—is highly needed.

Second, even though some scholars have demonstrated the influence of CSR on individual-level outcomes such as organizational commitment, organizational identification, and job satisfaction [16,17,18,19,20,21,22,23,24], the effects of CSR on creativity as well as its intermediating mechanisms have been relatively underexplored [12,13,15]. Although some studies [25,26] demonstrate a relationship between CSR and employee creativity, considering the importance of creativity in an organization, we believe that research examining the CSR–creativity link and its intermediating processes is relatively scarce. Insofar as creativity is an essential driver of a firm’s innovative capability, which is critical for survival in today’s extremely competitive business world [27,28], it is important to examine whether firms’ activities in social responsibility may increase the level of employees’ creativity.

Lastly, and more importantly, the previous works on corporate social responsibility have relatively underexplored the role of leadership behaviors in implementing CSR activities [13,15]. Since leaders not only substantially build employees’ perceptions, attitudes, and behaviors but also create the norms and rules in an organization [29,30,31,32,33,34], this paper suggests that leadership would play a significant role in the process of an organization’s CSR practices. Therefore, works that examines how leadership behaviors affect and interact with CSR activities are highly required.

To answer the calls described above, here, we investigate an intermediating process to explain how CSR influences employees’ creativity. Specifically, we propose an intermediating role of employees’ psychological safety between CSR and employees’ creativity. Psychological safety is defined as an employee’s perception that they are safe to take risks or confront tough issues in their organization [35]. The overall mediating structure of our argument is based on a group creativity model [36]. First, CSR stands to enhance psychological safety in employees by providing care and help in both direct and indirect ways. Based on the care and support, CSR activities are likely to increase employees’ psychological comfort by making them feel that their organizations are psychologically secure places [18,26,35,37].

In turn, enhanced levels of psychological safety in employees subsequently stand to boost creativity in employees. If an employee feels psychologically safe, then he or she may easily raise creative and novel ideas without fears of rejection or penalty by colleagues in their organizations [38,39]. Therefore, based on a group creativity model [36], we suggest that psychological safety functions as a critical mediator in the association between CSR and creativity.

More importantly, we expect that ethical leadership may play a role of critical moderator in the relationship between CSR and psychological safety. Ethical leadership is defined as “the demonstration of normatively appropriate conduct through personal actions and interpersonal relationships, and the promotion of such conduct to followers through two-way communication, reinforcement, and decision-making” ([40], p. 120). Considering that leaders are one of the most important sources who significantly build norms and rules in an organization [29,30,31,32,33,34], they would play a referent role which directly influences the perceptions and attitudes of employees toward their organization. Thus, we expect that the leadership behaviors may function as a critical environmental factor that moderates the influence of CSR activities.

To be specific, when the degree of ethical leadership is high in an organization, the followers are likely to consider that the organization’s moral value (that is reflected by CSR activities) and the leader’s one (that is reflected by ethical leadership) are consistent or congruent, making them feel that the moral or ethical values are certainly realized in the form of organizational norms or systems. Then, the employees would come to a conclusion that the moral values reflected by the moral practices (i.e., CSR) are authentic and trustworthy, eventually positively reacting to the CSR activities. A high level of ethical leadership may substantially amplify the positive impact of CSR on the employee’s perception such as psychological safety. In other words, ethical leadership would function as a positive moderator in the relationship between CSR activities and psychological safety. In contrast, when the level of ethical leadership is low, employees may feel confused about which values are dominant in the organization as well as how to react to the moral issue. Then, they may doubt whether the organization’s CSR practices are authentic and trustworthy. It ultimately decreases the positive impact of CSR on psychological safety. Thus, we argue that the degree of ethical leadership would moderate the CSR–psychological safety link.

In summary, the primary aim of our research was to investigate the effect of CSR practices on employees’ creativity, focusing on the intermediating process (i.e., mediator and moderator) in the link. We believe that this paper can positively contribute to CSR literature based on the following reasons. First, this paper examines the impact of CSR on micro-level outcomes such as employees’ creativity. Second, this study attempts to delve into the intermediating factors in the relationship. Third, we investigate the important role of leadership in translating CSR activities into positive responses of employees. Lastly, this paper can contribute to the enrichment of positive psychology literature [41]. We demonstrate that positive environments (e.g., CSR activities) create positive outcomes (e.g., creativity in employees) by boosting positive mindsets in employees (e.g., psychological safety).

## 2. Theory and Hypotheses

### 2.1. CSR and Psychological Safety

We suggest that CSR may increase the level of psychological safety in employees. Based on the basic concept of CSR [1,2,3,4,5], this paper regards that CSR practices include various charitable giving, investment, and services for internal stakeholders (such as employees) and external stakeholders (such as local communities, the natural environment, and customers) [18]. Through the altruistic behavior and support of organizations, employees may perceive that their organizations are charitable and trustworthy. Specifically, we suggest the influence of discrete aspects of CSR on psychological safety in employees as follows.

First, CSR for employees includes various practices for employees such as training, education, and safety programs [19]. Through such practices, employees may feel that they are cared for and treated as valuable members of their organizations. These types of perceptions allow employees to feel safe and protected by their organizations [18,19,20,21,22,23,24,25,26]. When employees feel supported and protected by their organizations, they develop psychological resources that enable them to show their true selves without fear of being judged, censored, or rejected [38].

In addition, through various programs and continuing education to enhance employees’ work-related abilities, an organization can increase employee levels of self-efficacy and/or competence. In turn, employees may feel that they have sufficient capacity to express their own opinions about work-related issues for the sake of solving various problems in their organizations [38]. Through these processes, CSR for employees may directly enhance psychological safety in employees.

Second, CSR activities for local communities may increase levels of employee psychological safety in indirect ways. Companies with high levels of CSR for their community are likely to emphasize the importance of the firm’s social responsibilities to various members in the community (e.g., manufacturers, suppliers, consumers, government organizations, non-governmental organizations, and the socially disadvantaged), as well as to encourage employees to spontaneously help members of the community [24]. Given that employees are also important members of the community, CSR activities for the community cause employees to perceive that they are cared for and supported by their organizations. In turn, these positive feelings in employees may contribute to sufficient levels of psychological safety in employees for them to be themselves in organizations [35].

Third, CSR activities for the environment may indirectly enhance levels of psychological safety in employees. When a firm actively implements CSR practices for the environment, employees in the firm may perceive their firm as a moral agent, contributing to the sustainability of the earth [6]. Employees’ perceptions of their organizations as ethical may decrease their levels of uncertainty and anxiety about various uncomfortable issues in the organization, making them feel less vulnerable [42]. Furthermore, if employees perceive that an organization is moral, then they are likely to believe that the organization is fair [43]. These kinds of positive influences, such as reduced negative emotions (e.g., anxiety) and increased belief in fairness, originate in CSR activities and facilitate employee perceptions that their organizations are reliable and trustworthy. Subsequently, these perceptions increase employees’ levels of psychological safety.

Lastly, CSR practices toward customers may increase employees’ levels of psychological safety in indirect ways. Corporate social responsibility (CSR) for customers indicates a company’s efforts to secure consumer rights beyond legal demands and practices that focus on maximizing customer satisfaction [24]. If an organization actively implements socially responsible practices for customers by prioritizing customers’ benefits above the interests of the organization itself, then employees in the organization may perceive the organization as ethical and trustworthy. From the viewpoint of employees, it is admirable for a firm to defer its own interests for the sake of customers. Thus, customer-centric CSR practices are considered to be moral activities. An employee’s trust in his or her organization, which originates in the organization’s morality (i.e., CSR activities), may cause them to believe that they are affiliated with a safe organization. In addition, employees tend to consider themselves as “internal customers” of their firms. Previous marketing literature has proposed that employees, as internal customers, function as the producers of goods or services in fulfilling the needs of end-customers [37]. Thus, when an organization implements CSR activities for customers, employees are likely to expect the organization to treat them as well as the organization treats external customers. This stands to enhance employees’ levels of psychological safety.

**Hypothesis** **1** **(H1).** *CSR is positively related to employees’ psychological safety.*

### 2.2. Psychological Safety and Employee Creativity

Existing studies have found that employees’ perceived psychological safety increases their levels of creativity [38,44]. Higher levels of creativity in employees result in the generation of new, original, and practical ideas associated with superior goods, services, and processes [45].

From the perspective of employees, building and expressing new ideas is closely associated with taking risks in an organization insofar as novel ideas, by nature, are necessarily accompanied by the possibility of failure [38,44]. Therefore, it is very difficult for employees to attempt not only to generate new ideas but also to actually implement new ideas in an organization. In other words, in order for employees to implement new ideas, they must believe that they are safe to make mistakes [35,44]. Employees with psychological safety in the workplace tend to raise questions about existing conventional thoughts and behaviors, as well as suggest new ways of solving various problems at work. Thus, employees’ perceptions of psychological safety may function as one of the most important factors in building creativity in organizations. Some previous studies on the relationship between psychological safety and creativity have empirically bolstered this argument [38,39]. Thus, we suggest the following hypothesis.

**Hypothesis** **2** **(H2).** *Psychological safety in employees is positively associated with employee creativity.*

### 2.3. Mediating Role of Psychological Safety between CSR and Employee Creativity

**Figure 1:**
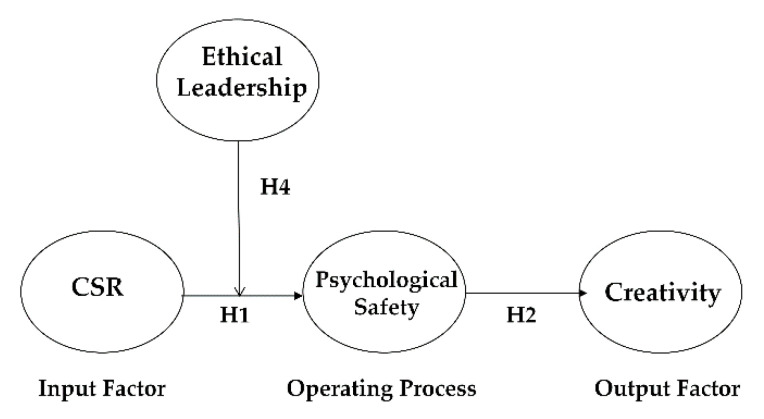
Theoretical model.

As described above, we propose that psychological safety in employees mediates the CSR–employee creativity link. Corporate social responsibility (CSR) enhances employees’ creativity by raising levels of employees’ psychological safety. To provide an overarching perspective based on solid theoretical ground, we rely on a group creativity model [36] that consists of input factors, operating processes, and output factors. The framework suggests that input factors (e.g., group member variables, group structures, group climate, and external demands) are likely to build creative outputs (e.g., individual-level or group-level creativity) by influencing various operating processes (e.g., cognitive, motivational, and social processes). Considering that CSR activities may function to inform critical group norms or group climate, CSR is considered an input factor. As such, the input factor positively affects employees’ cognitive processes such as psychological safety, eventually boosting creative outputs such as employees’ levels of creativity. Taken together, we propose that employees’ psychological safety mediates the CSR–employee creativity link. We form hypotheses as follows (also see Figure 1).

**Hypothesis** **3** **(H3).** *Psychological safety in employees mediates the relationship between CSR and employee creativity.*

### 2.4. Moderation Effect of Ethical Leadership in the CSR–Psychological Safety Link

Among the various behaviors of leaders which contribute to forming the norms and standards in an organization, in the present study, we focus on the ethical behaviors of leaders. In this paper, we propose that ethical leadership would function as an important contextual factor which moderates the association between CSR and psychological safety. To develop our arguments more logically, we provide the following reasoning.

Organizational scholars have noted that leaders play a critical role in creating employees’ perceptions, attitudes, and behaviors in an organization [46]. From the perspective of employees, their leader functions as a referent figure in forming their perceptions and attitudes toward rules or norms about what adequate behaviors are in the organization [30]. In other words, leaders function as (a) a primary source of information about what kind of roles their followers are expected to fulfil [34] and (b) a role model who determines what kind of behaviors are proper and requested from their followers as established norms and standards in the organization [31]. Therefore, we can expect that the leader’s behaviors can be an important environmental factor which creates norms or standards about proper work behaviors. In an organization, employees tend to find signals or cues not only to decrease uncertainty but also to increase predictability in their environments [29]. Thus, they are likely to be dependent on information which originates from their observations of their leader’s leadership behaviors [47]. Specifically, we expect that when the level of ethical leadership is high, the positive influence of CSR on psychological safety may be boosted. Considering that leaders function as a referent figure in creating employees’ perceptions of and attitudes toward rules or norms about what adequate behaviors are in the organization [30], the followers may perceive that the moral value of the organization (i.e., CSR activities) and the leader’s one (i.e., ethical leadership) are congruent. In that situation, employees may firmly believe that the ethical values which both their organization and leader pursue are certainly realized in the form of organizational practices or systems (e.g., CSR activities). From the perspective of the employees, the moral values which are reflected by moral practices (i.e., CSR activities) are reliable and authentic, and trust in the authenticity is likely to encourage the followers to positively react to the CSR activities by forming positive perceptions (i.e., psychological safety). Through the processes, the positive impact of CSR practices on psychological safety would be bolstered.

In contrast, when the leader exhibits a low level of ethical leadership, employees may perceive that the moral values that their organization pursue and the ones that their leader pursue are not consistent. In this situation, they are likely to be confused about which values are dominant in the organization as well as how to react to the situation. Then, they may doubt whether the moral values that are reflected in CSR practices are authentic and reliable. Then, the employees would not trust the authenticity of the moral practices, eventually negatively or cynically reacting to the CSR activities. Therefore, through the processes, the enhancing influence of CSR on employees’ psychological safety may be eroded. In conclusion, we propose the following hypothesis.

**Hypothesis** **4** **(H4).** *Ethical leadership would positively moderate the CSR–psychological safety link.*

Taken together, we suggest that CSR enhances employees’ creativity through a mediating effect of psychological safety. Moreover, ethical leadership positively moderates the association between CSR and psychological safety.

## 3. Research Methodology

### 3.1. Participants and Procedure

Using an online survey system, we collected survey data from currently working Korean employees across three different time points. The processes were conducted by one of the largest online research firms in South Korea, which is known to have about 1,300,000 panelists. By applying a random sampling method through providing an online link, the research company randomly selected 520 respondents who currently work in an organization among the 1,300,000 panelists. Then, the experts in the research firm directly contacted them to ask for permission of participation in our survey. The company provided the participants with a reward for their participation in the form of cash. The random sampling method may contribute to decreasing the biases due to employees’ characteristics that could influence the results of our research (e.g., gender, tenure, position, education, and industry type), eventually reducing the possibility of sampling bias. By virtue of the online system’s operating functions, we were able to track who responded to our survey, confirming that participants from time point 1 to time point 3 were the same.

At time point 1, a total of 512 participants responded to the initial survey. At time point 2, 378 employees participated in the second survey following the first survey. At time point 3, 335 employees responded to our third and final survey. The time interval between each time point was four or five weeks. Our survey system was opened for two or three days each at each time point to provide enough time for the participants to respond. When the system was open, the participant could access it whenever he or she wanted. After collecting data, we eliminated any missing data. Ultimately, data from 311 employees were utilized in our analysis. The characteristics of the participants are shown in Table 1.

### 3.2. Measures

Because the original items were developed in English, we translated them into Korean. After that, bilingual researchers back-translated the items. All research variables were measured by multi-item scales with a five-point Likert scale (with scores ranging from 1 = strongly disagree to 5 = strongly agree). We calculated the internal consistency of all variables using Cronbach’s alpha coefficients.

#### 3.2.1. CSR (Time Point 1, Collected from Employees)

We measured levels of CSR in each organization by adapting 12 items from the work of Farooq and his colleagues [19], originating in Turker’s CSR scale [24]. Although Turker’s original scale combined the environment dimension of CSR with the community dimension of CSR, the empirical work of Farooq and his colleagues splits the two dimensions by considering traditional and widely accepted perspectives on CSR [3]. Thus, our scale consists of four domains including environment, community, employee, and customer dimensions. Each of the selected four dimensions contains three items and represents corresponding stakeholders in social responsibility. For the environment dimension, sample items were “Our company participates in activities which aim to protect and improve the quality of the natural environment”, “Our company implements special programs to minimize its negative impact on the natural environment”, and “Our company targets sustainable growth which considers future generations”. For the community dimension, sample items were “Our company contributes to campaigns and projects that promote the well-being of society”, “Our company emphasizes the importance of its social responsibilities to society”, and “Our company actively participates in voluntary donations to charities and non-governmental organizations”. For the employee dimension, sample items were “Management at our company is primarily concerned with employees’ needs and wants”, “Our company policies encourage employees to develop their skills and careers”, and “Our company supports employees’ growth and development”. For the customer dimension, sample items were “Our company respects consumer rights beyond legal requirements”, “Our company provides full and accurate information about its products to its customers”, and “Customer satisfaction is highly important for our company”. These items have been utilized in previous studies conducted in the South Korean context [47,48,49]. By considering the structural position of CSR, we collected responses to these items at time point 1. The reliability coefficient in this study was 0.90.

#### 3.2.2. Psychological Safety (Time Point 2, Collected from Employees)

We measured psychological safety by using four items from a psychological safety scale developed in the work of Edmondson [35]. This scale measures followers’ perceptions of psychological safety. Sample items were “It is safe to take a risk in this organization”, “I am able to bring up problems and tough issues in this organization”, “It is easy for me to ask other members of this organization for help”, and “No one in this organization would deliberately act in a way that undermines my efforts”. These items have been utilized in existing empirical works conducted in the South Korean context [50]. The reliability coefficient in this study was 0.78.

#### 3.2.3. Creativity in Individual Employees (Time Point 3, Collected from Immediate Supervisors of Employees)

We measured employees’ creativity by adapting four items from a creativity scale developed by Tierney, Farmer, and Graen [51]. Based on previous research [50], an immediate supervisor of each participant rated the level of the participant’s creativity at work. To provide an adequate level of validity, we used items that have been utilized in previous empirical research [50]. Sample items were “This employee seeks new ideas and ways to solve problems”, “This employee demonstrates originality at his or her work”, “This employee tries out new ideas and approaches to problems”, “This employee generates novel but operable work-related ideas”, and “This employee is a role model of creativity in an organization”. The reliability coefficient of this study was 0.89.

#### 3.2.4. Ethical Leadership (Time Point 1, Collected from Employees)

Ethical leadership was assessed by the seven items of the ethical leadership scale by Brown and his colleagues [40]. In this paper, we utilized these items, including “My leader disciplines employees who violate ethical standards”, “My leader conducts his/her personal life in an ethical manner”, “My leader discusses business ethics or values with employees”, “My leader sets an example of how to do things the right way in terms of ethics”, “When making decisions, my leader asks what is the right thing to do”, “My leader listens to what employees have to say”, and “My supervisor can be trusted” (Cronbach’s alpha = 0.89).

#### 3.2.5. Control Variables (Time Point 2, Collected from Employees)

Based on existing studies [52,53], we utilized employee characteristics including gender, position, education level, tenure, and industry type as control variables because such factors may be associated with employees’ creativity.

### 3.3. Analytical Approach

For baseline statistics, a correlation analysis was conducted on our data. Considering that the hypothesized model of our research contains multiple variables, we conducted SEM analysis to test the moderated mediation model and obtain the fit indices of the model [54]. As an estimation method, we utilized the maximum likelihood (ML) procedure. Considering a suggestion from the work of Anderson and Gerbing [55], this paper takes a two-step approach consisting of the measurement model and the structural model. To assess the adequacy of the model fit, this paper utilizes several goodness-of-fit indices including a comparative fit index (CFI), the Tucker–Lewis index (TLI), and a root mean square error of approximation (RMSEA). Adequate fit indices are indicated by CFI and TLI values greater than 0.90 and an RMSEA value less than or equal to 0.06 [56].

## 4. Results

### 4.1. Descriptive Statistics

To obtain basic insights from the data, we calculated descriptive statistics. All of the descriptive statistics are shown in Table 2.

### 4.2. Measurement Model

We performed confirmatory factor analyses (CFAs) for all 23 items in order to examine the goodness-of-fit of the measurement model. Because three psychometric constructs (i.e., CSR, employees’ psychological safety, and ethical leadership) are incorporated in our research model, the discriminant validity of the three variables was identified. The three-factor model turned out to show a good fit with the observations (χ2 (df = 210) = 356.42; CFI = 0.959; TLI = 0.951; RMSEA = 0.047). Additionally, we conducted chi-square difference tests by sequentially comparing the three-factor model to the two-factor model (which combines CSR and ethical leadership into the same factor, χ2 (df = 212) = 697.69; CFI = 0.864; TLI = 0.838; RMSEA = 0.086) and the single-factor model (χ2 (df = 213) = 864.50; CFI = 0.818; TLI = 0.783; RMSEA = 0.099). The results of the chi-square difference tests demonstrated that our three-factor model has the best fit among the alternative models. The results confirmed the distinctiveness of the three variables (see Table 3).

### 4.3. Structural Model

#### 4.3.1. Results of Mediation Analysis

**Figure 2:**
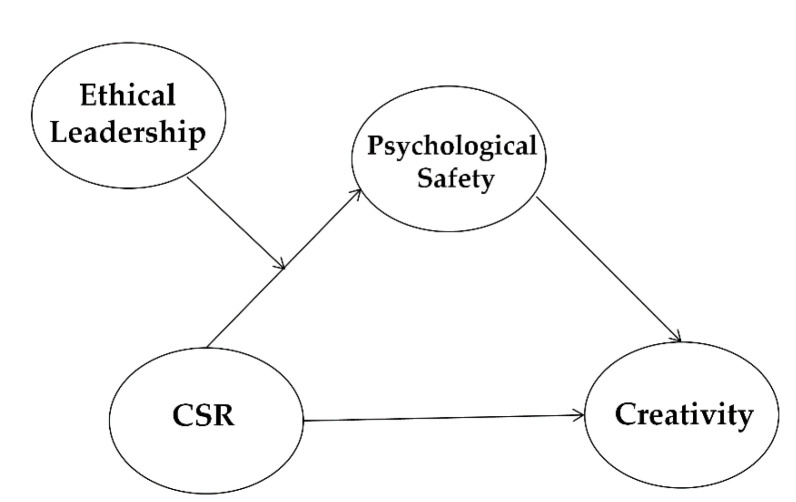
Alternative structural model.

To find the best model, we conducted SEM analyses and a chi-square difference test among all alternative models including the full mediation model and partial mediation model (see Figure 2). The partial mediation model is the same as the full mediation model except for including the path from CSR to creativity. The fit indices of all the alternative models were adequate. Results of the chi-square difference tests suggested that the partial mediation model (χ2 (df = 124) = 222.70; CFI = 0.949; TLI = 0.930; RMSEA = 0.051) has the better fit compared to the full mediation model (χ2 (df = 125) = 228.96; CFI = 0.946; TLI = 0.926; RMSEA = 0.052). This result indicates that CSR affects the level of employees’ creativity both directly and indirectly.

**Figure 3:**
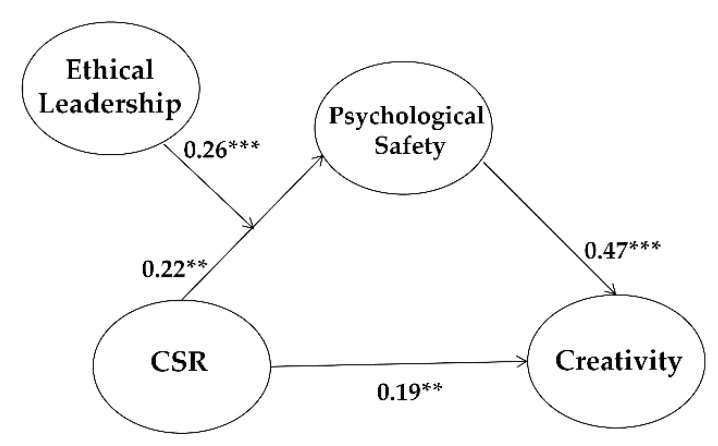
Final structural model. Standardized coefficients are presented. ** *p* < 0.01, *** *p* < 0.001.

Control variables including gender, position, tenure, industry type, and education level turned out to be statistically non-significant. Incorporating the control variables, our model shows supporting results for all of the hypotheses. CSR increases psychological safety (β = 0.22, *p* < 0.01), thus supporting Hypothesis 1, and psychological safety also enhances employees’ creativity (β = 0.47, *p* < 0.001), thus supporting Hypothesis 2 (please see Figure 3). Furthermore, the direct, indirect, and total effects of paths from CSR to levels of creativity in employees are provided in Table 4.

#### 4.3.2. Bootstrapping

Bootstrapping analyses were conducted by utilizing a sample of 10,000 [56] to test Hypothesis 3. Indirect mediation effects are significant at the 5% level if the 95% bias-corrected confidence interval (CI) for mean indirect mediation effects does not include zero [57]. Our results showed that the bias-corrected CI for the mean indirect effects on the paths did not include zero (95% CI = [0.03, 0.34]). Thus, we can conclude that Hypothesis 3 was supported.

### 4.4. Results of Moderation Analysis

The moderation influence of ethical leadership on the relationship between CSR and psychological safety was evaluated using the moderated mediation model. To make an interaction term, we conducted a mean-centering procedure. Centered variables not only estimate the interaction terms in an efficient way but also decrease multi-collinearity among the variables [58].

**Figure 4:**
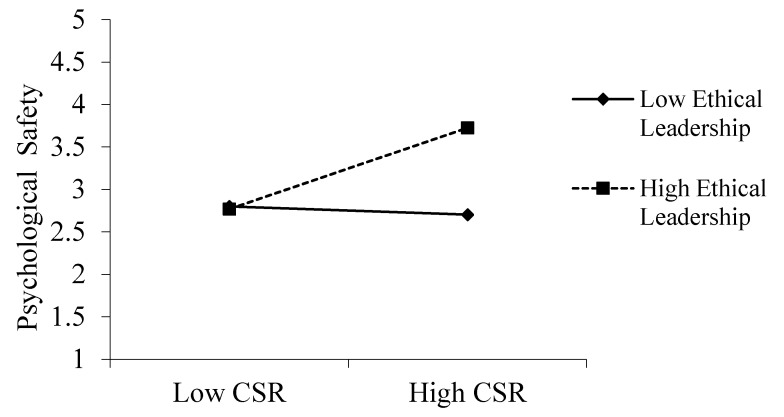
Moderating effect of ethical leadership in the corporate social responsibility (CSR)–psychological safety link.

The coefficient value of the interaction term (β = 0.26, *p* < 0.001) was statistically significant, indicating that ethical leadership positively moderates the CSR–psychological safety link. It indicates that when the degree of ethical leadership is high, the positive influence of CSR on psychological safety is increased, thus supporting Hypothesis 4(Please Figure 4).

## 5. Discussion

Using three-wave time-lagged data, we revealed that employees’ psychological safety functions as an intermediating mechanism in the association between CSR and creativity. We also found that ethical leadership positively moderates the CSR–psychological safety link. We believe that our research can contribute to the existing studies since we concentrate on the internal mechanisms of CSR by investigating mediating and moderating factors which explain why and when CSR may enhance creativity. In this following part, we discuss the theoretical and practical implications of this research. We also point out the limitations and suggest some of the possible future research directions.

### 5.1. Theoretical Implications

We believe that our research stands to contribute to CSR literature in a theoretical way by suggesting some important implications. First, we attempted to reveal the importance of internal stakeholders’ reactions toward CSR. Existing studies have not paid sufficient attention to the critical roles of employees’ perceptions, attitudes, and behaviors in translating CSR practices into organizational outcomes. The majority of the existing research takes an externally oriented approach to explain the association between CSR and organizational-level outcomes [1,13,15]. By focusing on employees’ reactions toward CSR in the form of individual-level outcomes (i.e., levels of psychological safety and creativity in employees), we emphasize the importance of the micro-foundations of CSR.

Second, we investigated the effects of CSR on levels of creativity in employees. Despite its theoretical and practical importance, employee creativity has not drawn nearly enough attention from CSR researchers. Previous research has manly focused on employees’ organizational commitment, organizational identification, and job satisfaction [16,17,18,19,20,21,22,23,24]. Considering that creativity in employees is a key building block for innovation in firms [27], our attempt to elucidate the CSR–creativity link may positively contribute to CSR literature.

Third, by delving into underlying processes in the CSR–employee creativity link, we revealed that psychological safety in employees functions as a crucial mediator to translate CSR activities into levels of employee creativity. This approach may contribute to resolving inconsistent findings regarding the association between CSR and organizational outcomes [12,13,14].

Fourth, this paper showed that leaders function as a critical contextual factor to explain the CSR–creativity link. Considering that employees tend to be influenced by the leaders’ thoughts, words, and behaviors when they form perceptions on what behaviors are appropriate or allowed in their organization [30], we expect that the leaders play a referent role which directly influences the implementation of various practices such as CSR. In other words, the leaders’ attitudes or behaviors may moderate the impact of CSR. Specifically, we demonstrated that the positive influence of CSR on psychological safety is strengthened by a high level of ethical leadership.

Lastly, we believe that this paper contributes to the enrichment of positive psychology literature. From the perspective of positive psychology, we demonstrated that positive environments (e.g., CSR activities) yield positive outcomes (e.g., creativity in employees) by facilitating positive mindsets in employees (e.g., psychological safety). This is meaningful for the promotion of healthy organizations in today’s extremely competitive business environments [41].

### 5.2. Practical Implications

This paper provides some practical implications for members of organizations, including top management teams, leaders, and employees. First, based on our findings that CSR activities enhance levels of creativity in employees, this paper suggests that CSR can be regarded as an effective “investment” rather than merely a “moral duty.” Given that employee creativity is a significant force in boosting innovation in firms (thereby providing firms with a meaningful competitive advantage) [27], enhancing employee creativity through CSR activities emerges as a reasonable strategy for firms.

Second, by understanding underlying processes in the CSR–creativity link, top management teams or leaders can estimate whether or not the CSR activities of their firms are effective. Our research demonstrates that moral practices can enhance creativity in employees by increasing employee levels of psychological safety. Thus, if top management teams or leaders want to boost employees’ creativity by implementing CSR practices, they must carefully monitor both levels and changes in employees’ attitudes (i.e., psychological safety). For example, if the level of an employee’s psychological safety is not enhanced after conducting CSR activities, then it may be that the moral practices do not work adequately. By observing trends in underlying processes, managers can glean important information about the effectiveness of CSR.

Third, we suggest that top management teams should fully understand the critical role of ethical leadership in implementing CSR practices. Considering that leaders are one of the most important sources who significantly build norms and rules in an organization, the leader’s characteristics, such as ethical leadership, would critically influence employees’ perceptions or attitudes on CSR activities. If the level of ethical leadership is low, the positive influence of the moral practices on employees’ attitudes would be eroded. In other words, employees are likely to regard the level of ethical leadership as a practical gage to evaluate whether the moral values of their organization are authentic. Therefore, top management teams have to not only understand the fact that the increasing effect of CSR on psychological safety should be grounded on the degree of ethical leadership, but also try to cultivate it in the organization.

### 5.3. Limitations and Suggestions for Future Studies

Although this paper provides meaningful implications for both scholars and practitioners in business fields, it has some limitations to address. First, even though we used a three-wave time-lagged dataset, we cannot verify the causal inferences as the research suggests. Because this study only utilized a simplified cross-lagged panel model in which all research variables were measured at discrete time points only, changes in the mediating mechanisms (i.e., increases or decreases) were not captured. Thus, future studies should design longitudinal research to demonstrate true causality among research variables. Second, this paper does not consider the issue of potential alternative explanations for mediating effects. Specifically, this research does not consider competitor variables in psychological safety. Our finding that the direct path from CSR to creativity is significant may suggest that other mediators exist in the associations. In addition, the possibility exists that leadership-related variables, such as a leader’s listening role [59], may function as a more direct mediator by influencing levels of psychological safety in employees. Third, this paper is based on data from employees in South Korean firms only. Given that employees’ perceptions and attitudes tend to be significantly affected by their cultural background [60], we must be cautious in interpreting and applying our results to employees in other cultural environments.

Fourth, this paper cannot be free from common method bias because CSR and employee levels of psychological safety were measured by the same person. Although our dataset not only takes on a form of time-lagged structure but also has a dependent variable that was measured by the immediate supervisors of participating employees, this fundamental limitation must be acknowledged. Fortunately, the results of CFA showed that the variables in our research model are distinctive. However, future research should take this issue into account.

Sixth, considering that this research mainly utilizes data from a lot of office workers in big-size companies, caution should be taken when generalizing the results into other organizational contexts. Due to the restriction of the data-gathering process, we could not collect data by using a stratified sampling process. Thus, we acknowledge that the biased characteristics of occupation and firm size would influence the overall results. Future studies should gather data from employees with various occupations beyond office workers (e.g., sales and marketing workers, manufacturing workers, etc.) in various firm sizes to deal with the issue.

Lastly, this paper does not apply multi-level theories and perspectives. The structure of our dataset could not be multi-leveled because the managers who evaluated the levels of employee creativity did not provide ratings on more than one of their employees. Each employee asked a manager to evaluate his or her level of creativity directly. Because the independent variable of this research (i.e., CSR) is considered a group-level variable, future research should take a multi-level approach.

## 6. Conclusions

Based on a group creativity model, this research examined the impacts of CSR on creativity. Our results show that CSR practices increase levels of creativity in employees by enhancing their levels of psychological safety. Furthermore, we found that ethical leadership functions as a critical contextual factor in the CSR–psychological safety link. It indicates that employees’ psychological safety functions as an intermediating mechanism in translating CSR activities into organizational outcomes (i.e., employee’s creativity). Furthermore, leaders’ ethical characteristics would facilitate the positive influence of CSR practices in an organization. Although this paper has some theoretical and practical limitations as described above, we believe that these results will positively contribute to CSR literature as well as creativity literature by elucidating the underlying mechanism and its contingent factor of the variables. We sincerely request that future studies should consider and deal with the limitations of this paper.

## Figures and Tables

**Table 1:** Descriptive characteristics of the sample.

Characteristic	Percent
Sex	
Male	47.3%
Female	52.7%
Age (years)	
20s	21.2%
30s	25.4%
40s	26.1%
50s	27.3%
Education	
Senior high school and below	14.5%
Community college	20.3%
Undergraduate	58.2%
Postgraduate and above	7.1%
Position	
Staff	30.2%
Assistant manager	24.4%
Manager or deputy general manager	22.2%
Department/general manager or director and above	23.2%
Occupation	
Office workers	63.7%
Administrative positions	19.3%
Sales and marketing	6.4%
Manufacturing	4.5%
Education	1.6%
Other	4.5%
Tenure (months)	
Below 50	51.4%
50 to 100	19.0%
100 to 150	13.8%
150 to 200	5.5%
200 to 250	4.5%
Above 250	5.8%
Firm size	
Above 500 members	47.9%
300–499 members	12.2%
100–299 members	15.1%
50–99 members	6.4%
Below 50 members	18.3%
Industry Type	
Manufacturing	24.1%
Services	14.8%
Construction	12.9%
Information services and telecommunications	10.9%
Education	9.3%
Health and welfare	8.4%
Public service and administration	7.8%
Financial/insurance	3.9%

**Table 2:** Means, standard deviations, and intercorrelations of measures.

	Mean	SD	1	2	3	4	5	6	7	8
1. CSR	3.19	0.62	-							
2. Ethical leadership	3.12	0.67	0.49 **	-						
3. Psychological safety	3.10	0.64	0.32 **	0.33 **	-					
4. Creativity	3.17	0.64	0.32 **	0.25 **	0.43 **	-				
5. Gender	1.53	0.50	−0.16 **	−0.12 *	0.03	−0.04	-			
6. Position	2.71	1.62	0.12 *	0.08	0.08	0.16 **	−0.30 **	-		
7. Tenure (months)	93.10	95.02	0.22 **	0.09	0.09	0.12 *	−0.18 **	0.37 **	-	
8. Education	2.56	0.82	0.03	−0.03	−0.06	0.00	−0.08	0.10	0.08	^-^
9. Industry type	-	-	0.03	0.03	0.06	0.06	25 **	−0.01	0.00	0.05

Note: * *p* < 0.05. ** *p* < 0.01. For employee sex, males are coded as 1 and females as 2. For position, positions of general manager or above are coded as 5, deputy general manager and department manager as 4, assistant manager as 3, clerk as 2, and others below clerk as 1. For education, the level “below high school diploma” is coded as 1, “community college” as 2, “Bachelor’s” as 3, and “Master’s degree or higher” as 4.

**Table 3:** Chi-square difference tests among alternative measurement models.

	χ^2^	df	CFI	TLI	RMSEA	Model Comparison	Δdf	Δχ^2^	Preference
1-factor	864.50	213	0.818	0.783	0.099	1-factor vs. 2-factor	1	166.81	2-factor
2-factor	697.69	212	0.864	0.838	0.086				
3-factor	356.42	210	0.959	0.951	0.047	2-factor vs. 3-factor	2	341.27	3-factor

**Table 4:** Direct, indirect, and total effects of the final research model.

Model	Direct Effects	Indirect Effects	Total Effects
Internal CSR -> Creativity	0.187	0.100	0.288

All values are standardized.

## Data Availability

No new data were created or analyzed in this study. Data sharing is not applicable to this article.
